# The future of intensive care: the study of the microcirculation will help to guide our therapies

**DOI:** 10.1186/s13054-023-04474-x

**Published:** 2023-05-16

**Authors:** J. Duranteau, D. De Backer, K. Donadello, N. I. Shapiro, S. D. Hutchings, A. Rovas, M. Legrand, A. Harrois, C. Ince

**Affiliations:** 1 Department of Anesthesiology and Intensive Care, Bicêtre Hospital, Assistance Publique Hôpitaux de Paris (AP-HP), INSERM UMR-S 999, Paris-Saclay University, Le Kremlin-Bicêtre, France; 2 Department of Intensive Care, CHIREC Hospitals, Université Libre de Bruxelles, Boulevard du Triomphe 201, 1160 Brussels, Belgium; 3 Anaesthesia and Intensive Care Unit B, Department of Surgery, Dentistry, Paediatrics and Gynaecology, University of Verona, University Hospital Integrated Trust of Verona, Verona, Italy; 4 Department of Emergency Medicine, Beth Israel Deaconess Medical Center–Harvard Medical School, Boston, MA USA; 5 King’s College Hospital NHS Foundation Trust, London, UK; 6 Academic Department of Military Anaesthesia and Critical Care, Royal Centre for Defence Medicine, Birmingham, UK; 7 Division of General Internal and Emergency Medicine, Nephrology, and Rheumatology, Department of Medicine D, University Hospital Münster, Albert-Schweitzer-Campus 1, 48149 Münster, Germany; 8 Division of Critical Care Medicine, Department of Anesthesia and Perioperative Care, UCSF, San Francisco, USA; 9 Department of Intensive Care, Erasmus MC, University Medical Center, Rotterdam, The Netherlands

**Keywords:** Hemodynamic resuscitation, Microcirculation, ICU, Hand-held vital microscopes, Artificial intelligence

## Abstract

The goal of hemodynamic resuscitation is to optimize the microcirculation of organs to meet their oxygen and metabolic needs. Clinicians are currently blind to what is happening in the microcirculation of organs, which prevents them from achieving an additional degree of individualization of the hemodynamic resuscitation at tissue level. Indeed, clinicians never know whether optimization of the microcirculation and tissue oxygenation is actually achieved after macrovascular hemodynamic optimization. The challenge for the future is to have noninvasive, easy-to-use equipment that allows reliable assessment and immediate quantitative analysis of the microcirculation at the bedside. There are different methods for assessing the microcirculation at the bedside; all have strengths and challenges. The use of automated analysis and the future possibility of introducing artificial intelligence into analysis software could eliminate observer bias and provide guidance on microvascular-targeted treatment options. In addition, to gain caregiver confidence and support for the need to monitor the microcirculation, it is necessary to demonstrate that incorporating microcirculation analysis into the reasoning guiding hemodynamic resuscitation prevents organ dysfunction and improves the outcome of critically ill patients.

## Introduction

The core of hemodynamic resuscitation has traditionally focused on blood pressure and cardiac output; however, these measurements imperfectly reflect tissue perfusion. The recent emphasis on clinical signs of tissue perfusion such as capillary refill time and skin mottling is an important step toward a perfusion driven resuscitation. However, these types of skin perfusion assessment techniques are severely limited as these indices assess a relatively large volume of tissue and alterations in other microcirculatory beds may remain hidden using these techniques.

In patients in shock of various origins, an important number of studies have consistently demonstrated that persistent microcirculatory alterations are associated with organ dysfunction and mortality. More than 600 papers have highlighted the clinical relevance bedside monitoring of the microcirculation. This level of interest led to the publication in 2018 of guidelines for the assessment of sublingual microcirculation by the European Society of Intensive Care Society Task Force [1].

The future challenge is to transform microcirculation monitoring from an important research tool into an essential bedside monitoring technique used by clinicians to individualize hemodynamic resuscitation based on microvascular parameters. The purpose of this paper is to provide an update on the current state of microcirculatory monitoring in critically ill patients, and to present an approach for guiding therapy. We present a viewpoint on its potential role in the future of hemodynamic monitoring and on how it could influence the hemodynamic management of critically ill patients.

## Why is the study of the microcirculation essential to help guiding therapeutic strategy in ICU?

The two main determinants of the primary function of the microcirculation for oxygen transport are convection (e.g., the flow of oxygen-carrying red blood cells) and diffusion (e.g., the distance oxygen must travel from the red blood cell (RBC) to the cells). Parameters related to the convective (e.g., RBC flow rate) and diffusive (e.g., functional capillary density) capacity of the microcirculation are used to quantify the functional state of the microcirculation. Most hemodynamic strategies used in ICU focus on promoting blood flow and arterial oxygen transport (convection). However, achieving adequate diffusing capacity is also essential for optimal oxygen transport to the tissues, a variable that can only be measured by direct observation of the microcirculation. For example, the diffusive capacity of the microcirculation may be compromised during fluid therapy if increased RBC flow cannot compensate for dilution of RBC mass and if tissue edema induces increased diffusion distances between RBC and tissue cells, making it more difficult for oxygen to reach the latter. Understanding these two main components of oxygen transport to cells is essential to best guide hemodynamic strategies.

The analysis of the microcirculation allows clinicians to appreciate the behavior of the different constituents of the blood and their interactions with the endothelium and the glycocalyx. For example, its observation by hand-held vital microscopes (HVM) not only allows detailed quantification of the behavior of red blood cells directly responsible for oxygen transport to tissues, but also allows observation and quantification of the behavior of leukocytes [2]. Visualization of the microcirculation also allows for an indirect assessment of the integrity of the glycocalyx. Indeed, glycocalyx impairment allows a greater number of RBC to deviate, approach the endothelium and penetrate the permeable part of the glycocalyx layer. It is proposed to calculate this dynamic lateral movement of the RBC as an indirect inverse measure of the glycocalyx integrity (PBR, perfused boundary region) [3, 4].

**Fig. 1:**
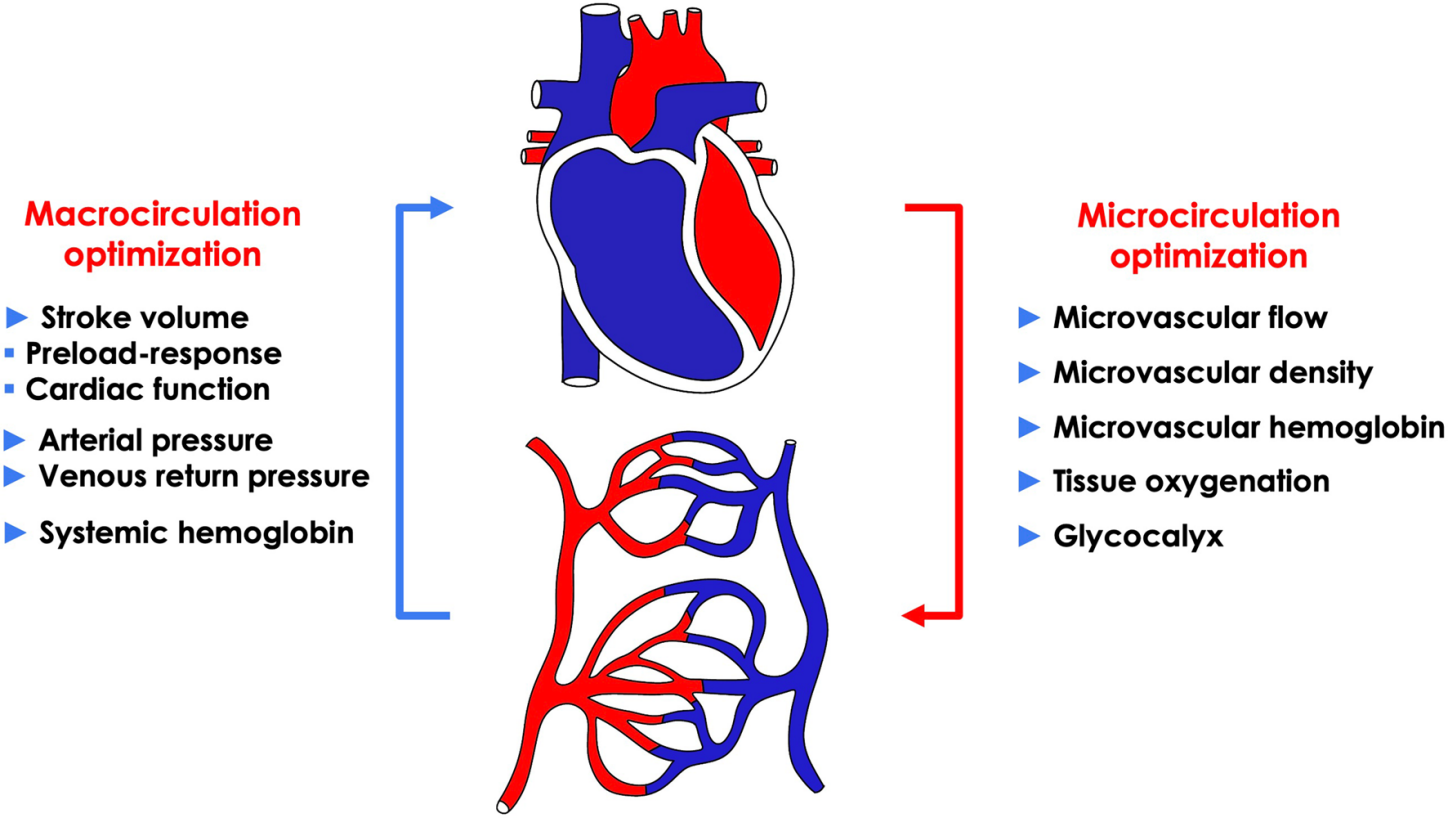
Different parameters of macrocirculation and microcirculation optimization

The goal of hemodynamic resuscitation is to meet the oxygen and metabolic needs of the various organs, which can only occur through optimization of the microcirculation (Fig. 1). We hope to achieve this goal through the optimization of macro-hemodynamic variables such as blood pressure and stroke volume (SV). But we never know if an optimization of microcirculation and tissue oxygenation is really achieved after macrovascular optimization. Decreases in microvascular flow and density are usually corrected by optimizing the macrocirculation, as there is hemodynamic coherence (i.e., harmony) between the macrocirculation and the microcirculation. On the other hand, optimization of macrocirculation may fail to improve tissue perfusion in the presence of alterations within the microcirculation. Since an impaired microcirculation occurs due to multiple factors which includes alterations in blood viscosity, endothelial dysfunction, glycocalyx degradation and/or microthrombi/microaggregates, many of these problems are not corrected by classic hemodynamic interventions (Fig. 1). Another risk is to over-optimize the macrocirculation in relation to the needs of the microcirculation and to end up with fluid overload or overuse of vasopressors that is often harmful in terms of tissue oxygenation. Clinicians are currently blind to what is happening in the microcirculation of organs, which prevents them from individualizing resuscitation by targeting the microcirculation. For example, Harrois et al. [5] found significant differences in renal cortical microcirculation recovery in patients with septic shock after macrovascular hemodynamic optimization. Indeed, in some patients, renal cortical microcirculation was satisfactory or even high, whereas in others, an alteration of this microcirculation persisted and was associated with the development of acute kidney injury (AKI). This result was confirmed by Watchorn et al. [6] who showed that the severity of AKI was related to the degree of renal cortical hypoperfusion independently of macrovascular optimization in patients in septic shock.

This is essential because clinical studies in different states of shock both in adult and pediatric patients have consistently shown that the persistence of microcirculatory alterations with lost of coherence between macrocirculation and microcirculation is predictive of organ failure and unfavorable outcomes in a more sensitive and specific manner than systemic hemodynamic and biological parameters [7–16]. Previous studies have demonstrated marked heterogeneity of microcirculatory flow in septic patients, with the presence of occluded capillaries next to perfused capillaries inducing microcirculatory shunting responsible for the decreased oxygen extraction capacity in sepsis [17]. In contrast to the reduction in oxygen extraction in sepsis, a recent study of the microcirculation response to COVID-19 identified an adaptive response of the microcirculation to increase its oxygen extraction capacity in response to COVID-19-induced hypoxemia [17]. This COVID-19-induced increase in microcirculatory oxygen extraction capacity was attributed to an increase in functional capillary density and capillary hematocrit. But the adaptive response may be impaired during hyperinflammation because of the inflammatory-induced alterations of the endothelium and glycocalyx and of a concomitant procoagulant state [18].

An other illustrative example of the potential interest in microcirculation assessment is the evaluation of the response to fluids. While a lot of emphasis has historically focused on the SV response to fluid infusion, the microcirculation represents a perhaps more element of the response in terms of tissue perfusion [19–22]. A study by Ospina et al. found that fluid administration can improve the microcirculation at early but not at later stages of sepsis [19]. Furthermore, Pottecher et al. [20] showed that a first bolus, but not a second bolus, of fluid improved the sublingual microcirculation independently of the SV increase in patients with septic shock [20]. In both trials, the microcirculatory effects were dissociated from the systemic effects. Pranskunas et al. [22] reported that patients who had impaired microcirculatory perfusion that improved with fluid therapy had an associated improvement in organ function, whereas patients who had normal microcirculatory perfusion initially or who failed to improve their microcirculation in response to fluids did not have an associated improvement in organ function. Such a distinction which could not be made by measurement of SV in this study [22]. Indeed, these findings highlight the importance of the microcirculation in the response to fluids and support the need to assess the microcirculation to guide fluid titration.

It is therefore necessary to integrate the analysis of the microcirculation in the reasoning guiding hemodynamic resuscitation to prevent organ dysfunction and improve the outcome of critically ill patients. Hemodynamic individualization based solely on macrocirculatory parameters is an incomplete view of hemodynamic optimization and the microcirculation must also be taken into account.

## How will we assess and analyze the microcirculation in ICU in the future?

There are a number of different methods to assess the microcirculation at the patient's bedside; all have strengths and future challenges (Table 1). It is important to consider that many advanced tools will not be accessible to low- and medium-outcome health care systems, and that the accessibility of microcirculation tools is important to consider for broad application of strategies.

**Table 1:** Strengths and challenges of microcirculation analysis methods in ICU

**Capillary refill time (CRT)**	
Technique	Pressure application to the fingertip for at least 10 s until the skin showed whitening. The time until return of baseline coloration after release of the pressure is measured with a chronometer (normal CRT ≤ 3 s)
Strengths	Simple and quickly measurable. Visual assessment. Easy team adhesion
Challenge for the future	Since it is a visual assessment, important to obtain objective and reliable measures of CRT
**Contrast-enhanced ultrasound (CEUS)**	
Technique	Ultrasound device with a probe appropriate to the region studied
	Uses gas microbubbles surrounded by a stabilizing envelope (phospholipid or protein envelope) of a size like that of red blood cells allowing them to cross the pulmonary capillary bed and reach the capillaries of the different organs. At the same time, their size is large enough that they do not cross the endothelium, making them true intravascular agents
	Microbubbles can be injected as a bolus or as a continuous infusion (with a rotating syringe pump). When a constant infusion is administered, a “destruction-replacement technique” can be used (interest of a baseline measurement)
	Quantitative analysis can be performed by different software. For each regions of interest (ROI), the software generates a time–intensity curve and calculates amplitude and time parameters which are proportional to blood volume and microvascular blood flow
Strengths	Can be used at the patient bedside. Availability of echography with specific software in ICU
	Analysis of the microcirculation and regional perfusion of deep organs
Challenge for the future	High variability of measurements
	Need for contrast with a specific cost
	Need for a shared perfusion protocol
**Hand-held vital microscopes (HVMs)**	
Technique	Direct noninvasive real-time visualization of capillary network
	Sublingual microcirculation is the most frequently studied microcirculation at the bedside
	Videos are analyzed with software to document changes in small blood vessels (blood vessels < 20 μm in diameter)
	Based on the software available
	Semiquantitative blood flow characteristics, as well as microcirculation flow index (MFI), total vessel density (TVD), perfusion and blood vessel ratio (PPV), and perfusion vessel density (PVD) are analyzed
	Quantitative per vascular diameter class analysis of vascular density, glycocalyx dimensions (PBR) and red blood cell velocity in static/dynamic state. Combining microvascular and glycocalyx variables allows the calculation of microvascular health score (MVHS)
Strengths	International consensus for video capture
	Large database validation of automated quantification of microvessel density and red blood cell velocity which can take the next steps toward real-time clinical application at the bedside
	Allows assessment of leukocyte behavior and glycocalyx integrity
Challenge for the future	Simplification of image acquisition and analysis
	Addition of Hb and SO_2_ measurements
	Measurements of local metabolism and/or redox states
	Setting clear microvascular targets
**Laser-Doppler flowmetry**	
Technique	Shift in light wavelength is proportional to the red blood cell velocity in the studied area
	Noninvasive measurement
	Expressed as arbitrary perfusion units (PUs)Simplification of image acquisition and analysis
Strengths	Skin laser Doppler coupled with local thermal challenge may provide a measure of microcirculatory reactivity
	Microcirculatory reactivity is decreased in patients with circulatory shock and has prognostic value
Challenge for the future	Impact of monitoring SDF with local thermal challenge on outcome in critically ill patients?
**Magnetic resonance imaging (MRI)**	
Technique	Several techniques available today, which can be combined into a single multiparametric MRI (phase contrast (PC-MRI), arterial spin labeling (ASL), diffusion weighted imaging (DWI) and blood oxygen level-dependent (BOLD) MRI
Strengths	Can help characterize the intensity of microvascular and oxygenation alterations in multiple organs (heart, brain and kidney) in a range of clinical scenarios
	Can also provide information to assess recovery from these alterations
Challenge for the future	Cannot be used to dynamically monitor the microcirculation in real time at the patient’s bed
	Need for radiological expertise
**Nailfold videocapillaroscopy (NVC)**	
Technique	Digital videocapillaroscope connected to analysis software. Semiquantitative score NVC abnormalities. An average score is calculated by analyzing 4 consecutive one-mm fields in the middle of the nail fold of each finger. The average scores of eight fingers are taken into account
Strengths	Noninvasive technique with standardization
Challenge for the future	Demonstrate the feasibility of the technique in ICU
	Need to develop an automated analysis of NVC images (with incorporation of red blood cell velocity)
**Near-infrared spectroscopy (NIRS)**	
Technique	Tissue oxygenation saturation (StO_2_) is the ratio of oxygenated to total tissue hemoglobin concentration ((oxyhemoglobin/(oxyhemoglobin + deoxyhemoglobin)) × 100%)
Strengths	Noninvasive and easy to use
	Thenar NIRS with a vascular occlusion test (VOT), Cerebral and renal NIRS
Challenge for the future	Clearly define the physiological significance of the NIRS-derived values
	Standardization of NIRS VOT (duration, level of inflation of cuff, timing between two inflations)
	Which target values should be reached?
**Plethysmography**	
Technique	Pulse co-oximetry continuously provides a noninvasive measure of peripheral perfusion, called perfusion index (PI)
	Peripheral PI is derived from the photoelectric plethysmography signal of pulse oximetry
	PI reflects the ratio of pulsatile and non-pulsatile light absorbance of the red and infrared light passing through the tissue
Strengths	Easy adherence by teams
	PI can be used to assess fluid responsiveness. Also allows for continuous noninvasive monitoring of hemoglobin concentration (SpHb) and oxygen reserve index (ORi)
	ORi monitoring anticipates SpO_2_ < 94% episodes and reduces the incidence of hypoxemia by giving the clinician additional time to act and optimize oxygenation and ventilation
Challenge for the future	Need to evaluate accuracy (bias) and precision (i.e., repeatability), but also in terms of the ability to identify trends
	Reproducibility of measurements using different devices/software (are PI measurements obtained by different devices identical?)
**Veno-arterial PCO**_**2**_** gap**	
Technique	Veno-arterial difference in the partial pressure of carbon dioxide (Pv-aCO_2_ gap)
Strengths	Reliable indicator of impaired tissue perfusion, whether the result of a global reduction in cardiac output or to microcirculatory abnormalities
	Does not track tissue dysoxia, unless related to low flow conditions
	Easily accessible and available. Can be included in diagnostic and therapeutic algorithms
Challenge for the future	Demonstrate that normalization of a Pva-CO_2_ difference has an impact on the outcome of patients in shock

**Fig. 2:**
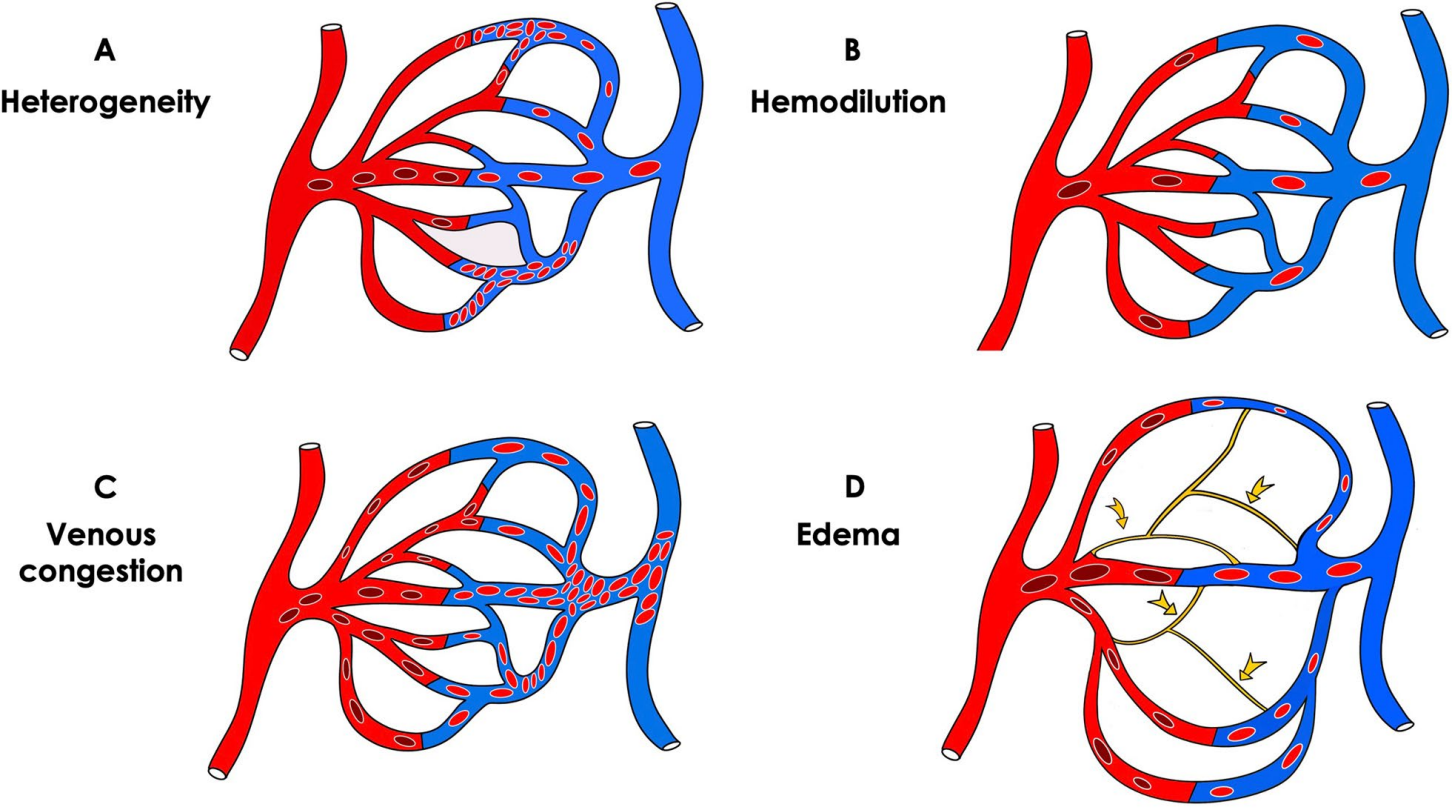
Illustration of the different types of microvascular alterations occurring despite macrovascular optimization of the macrocirculation. **A** Heterogeneous distribution, with perfused capillaries next to non-circulating capillaries, observed mainly in inflammatory and/or severe septic states. **B** Dilution of red blood cells occurring during hemodilution (for example in hemorrhagic shock during fluid resuscitation) and anemia. **C** Congestion due to increased venous pressure. **D** Tissue edema with increased oxygen diffusion distances

The recent introduction and validation of automated microcirculatory analysis software allowing point-of-care application of sublingual microcirculation-guided therapy is a significant step toward the introduction of routine use of HVM technology at the bedside [23, 24]. It provides quantitative microcirculatory functional parameters calculated from images including functional capillary density and RBC velocity allowing for the distinction between diffusive and convective alterations of the microcirculation (Fig. 2). The addition of new functional parameters such as capillary hematocrit, tissue RBC perfusion and the quantification of activated leucocytes provides even more information regarding the nature of microcirculatory alterations [24]. These variables were beneficial to the characterization of microcirculatory alterations in COVID-19 patients [17]. The microcirculation’s ability to increase its capillary-hematocrit-to-systemic-hematocrit ratio and FCD was only present in COVID-19 patients whose SOFA scores was less than 10. Conversely, no microvascular adaptive response was observed in COVID-19 patients with a SOFA score ≥ 10 [17]. The missing pieces in the actual evaluation of the microcirculation is the evaluation of microvascular O_2_ delivery and local metabolism. Different adaptations of optics can allow measurements of hemoglobin levels and O_2_ saturation in microvascular vessels. In addition, it is also feasible to assess local redox state of the mitochondria through the analysis of ultraviolet absorbance [25]. In future HVM may integrate these various optics, this would offer a unique opportunity to evaluate oxygen delivery and metabolism at the microvascular level, together with its consequences on mitochondrial function.

A further expansion of microcirculatory monitoring will occur when microcirculatory information is obtained from the microcirculation of organs themselves, as opposed to using the sublingual area as a proxy. Indeed, prior studies have assessed skin, conjunctiva, nail fold, rectal, stoma and vaginal microcirculations in various clinical conditions, although sublingual microcirculation is by far the most studied and clinically relevant microcirculatory bed to date. Even though several experimental studies have shown a coherence between sublingual and other organ surfaces, such as the intestines and kidney microcirculation [26, 27], it is conceivable that there are differences in behavior between the microcirculatory beds of different organ surfaces depending on the clinical circumstances [28, 29]. The inclusion of information regarding inflammatory activation of the microcirculation by observation of altered leucocyte kinetics [2], the presence of pathogens and/or the presence of microthrombi [17] is an interesting potential direction. Besides observing the different microcirculatory beds of the different organ surfaces, observation of the tissue cells and even subcellular structure may also provide more detailed information regarding the nature of tissue injury and organ function. This would require higher magnification HVM. The presence of a bubble under the HVM cap can cause additional magnification revealing individual parenchymal cells with membrane-to-membrane junctions and even making nuclei clearly visible [30].

An interesting technique for monitoring organ microcirculation at the patient’s bed is contrast-enhanced ultrasound (CEUS) which uses gas microbubbles surrounded by a stabilizing envelope (phospholipid or protein envelope) (Table 1). Different currently available software can perform this quantitative analysis. Renal CEUS has been proposed to quantify the renal microcirculation in patients under various conditions, such as renal transplantation [31, 32], or cardiac surgery with vasodilatory shock [5, 6, 33]. CEUS also holds potential to test the renal microvascular effects of fluid resuscitation and vasopressor therapy in ICU patients [5, 34]. The use of this technique with microcirculatory flow imaging is currently under study and remains reserved for clinical research at this time. Indeed, its use requires standardization to control the heterogeneity of the results, especially when using microbubble boluses.

Several techniques for the evaluation of peripheral perfusion are proposed (Table 1). Alterations in skin perfusion may occur before alterations in macrovascular hemodynamic variables, and prior data have demonstrated that the persistence of these alterations despite macrovascular optimization is associated with higher mortality [35]. Data support that accurate assessment of capillary refill time (CRT) is at least as useful as blood lactate level as a resuscitation target [36]. In a recent prospective study, skin blood flow (SBF) using skin laser Doppler was impaired in patients in circulatory shock, even though patients were hemodynamically stabilized [37]. SBF was lower in non-survivors than in survivors with a persistently blunted SBF response to thermal challenge test. Baseline SBF and SBF thermal challenge were both better predictors of ICU mortality than blood lactate, Scvo_2_, CRT and peripheral perfusion index (PPI) [37]. These peripheral perfusion assessment techniques are exciting tools for the future. The challenge now is to demonstrate that they are reproducible and can guide resuscitation and reduce organ dysfunction. In addition, the relation between these skin perfusion variables and the microcirculation and function of essential organs such as the kidney heart and brain still requires further study.

Near-infrared spectroscopy (NIRS) (Table 1) has been studied as a noninvasive methodology for assessing tissue oxygenation since the 1970s [38]. It has been assessed as a potential monitoring tool during surgery, particularly in cardiac surgery, or in ECMO patients to assess brain oxygenation [39, 40]. It has also been used to assess muscle tissue oxygenation in sepsis [41, 42] and traumatic hemorrhagic shock [43]. Associated with a vascular occlusion test (VOT), NIRS proposes an analysis of dynamic parameters of tissue O_2_ extraction and microvascular reactivity. A slower recovery of StO_2_ during the reperfusion phase is an independent predictor of mortality in patients with sepsis [44].

Magnetic resonance imaging (MRI) (Table 1) allows for the assessment of tissue perfusion and oxygenation [45–47]. There are several techniques available today, which can be combined into a single multiparametric MRI (phase contrast (PC-MRI) and include: arterial spin labeling (ASL), diffusion weighted imaging (DWI) and blood oxygen level-dependent (BOLD) MRI). Unfortunately, it is not feasible to use MRI to dynamically monitor the microcirculation at bedside.

The monitoring of brain microcirculation is a challenge due to its inaccessibility. However, the retina is considered a window to the brain [48] and retinal oximetry is a potential bedside technique [49] to assess brain microcirculatory dysfunction. Future HVM with long focal distances hold potential to directly observe the retinal microcirculation which could serve as an indirect measure of brain microcirculation.

## What evidence is needed to facilitate adoption of microcirculation analysis as a routine part of ICU therapeutic management?

**Fig. 3:**
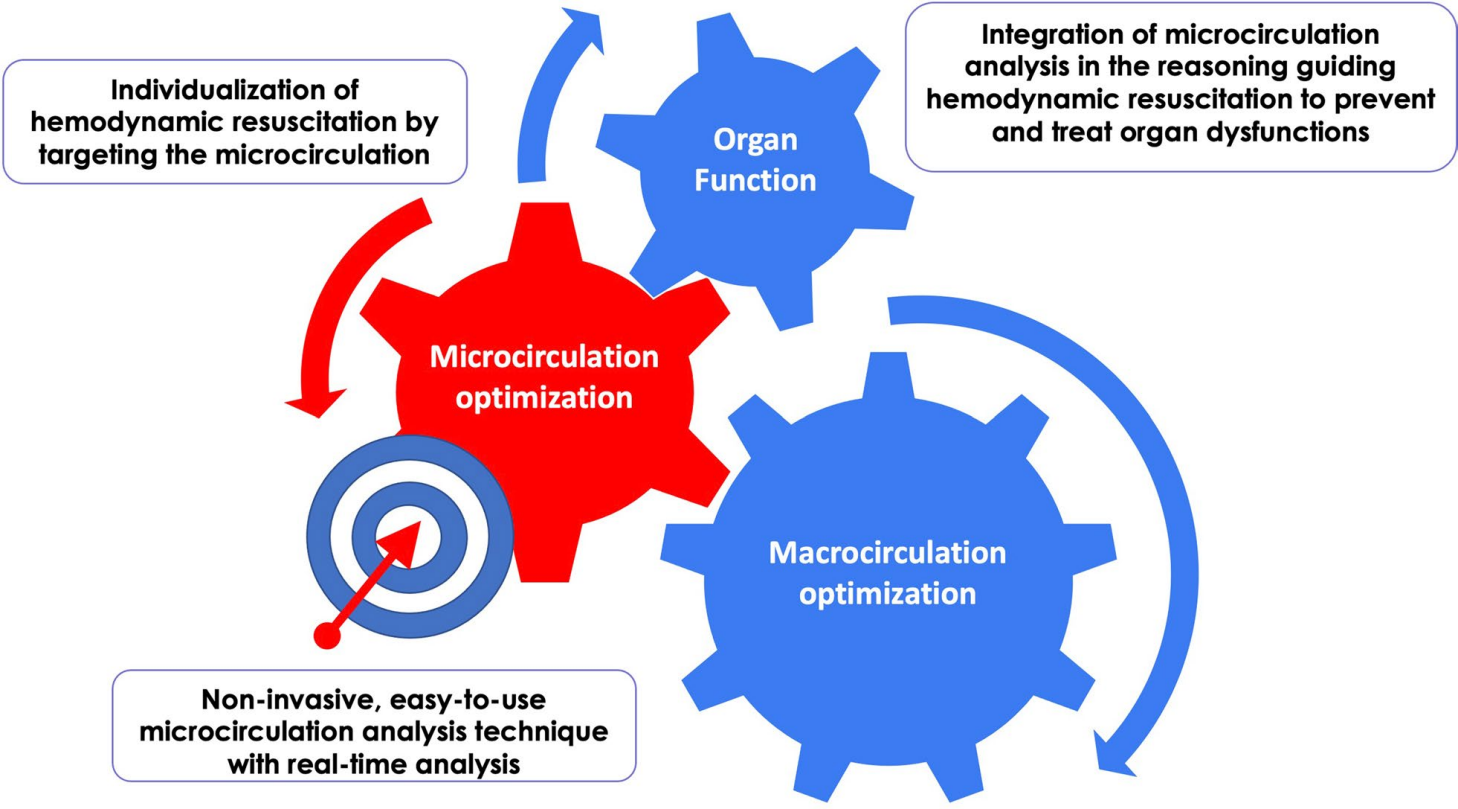
The challenge for the future of microcirculation monitoring in ICU

In order to establish microcirculation analysis as a standard of care, it is necessary to demonstrate that the integration of microcirculation analysis has an impact on the prevention and treatment of organ dysfunction (Fig. 3). It is also essential to have a microcirculation analysis device that is relevant (at best convective and diffusive microcirculatory analysis) and easily usable at the bedside (Fig. 3). Ideally, this would entail the combination of bedside equipment with software that performs reliable and immediate data analysis. Initial application of artificial intelligence shows promise as a technique to identify specific patterns of microvascular alterations that could identify microcirculatory impairment and guide therapy in future applications.

Very few studies have used microcirculation-targeted resuscitation. The ANDROMEDA-SHOCK trial [36, 50] suggested that a microcirculation-guided strategy based on CRT, as a surrogate parameter for microcirculatory perfusion, might limit organ failure and lower mortality compared to a lactate-targeted one. However, mortality was high in this study, and further studies are needed to develop more fine-grained microcirculation assessment approaches that provide a better understanding of the complexity of microvascular damage. Indeed, microvascular damage is not limited to hypoperfusion, and it is important to detect endothelial damage, glycocalyx damage and imbalances in antiprocoagulant balance (shock-induced endotheliopathy). Still, with the goal of impacting mortality, a focus on patients in whom microvascular alterations persist despite macrocirculatory hemodynamic optimization is needed. Once these patients are identified, we must then develop and test treatments that target restoration of the microcirculation. In this sense, the I-MICRO RCT [51] proposes to test the impact of ilomedin (a prostacyclin analogue with vasodilatory and antithrombotic properties) on organ failure in septic shock patients with persistent microcirculatory disorders (assessed by mottling score and/or skin recoloration time) despite hemodynamic optimization. In the future, there is a need to design studies that integrate the implementation of microcirculation-guided resuscitation in hemodynamic optimization, and to identify microvascular-targeted treatment and strategies that improve outcomes in critical care patients.

**Fig. 4:**
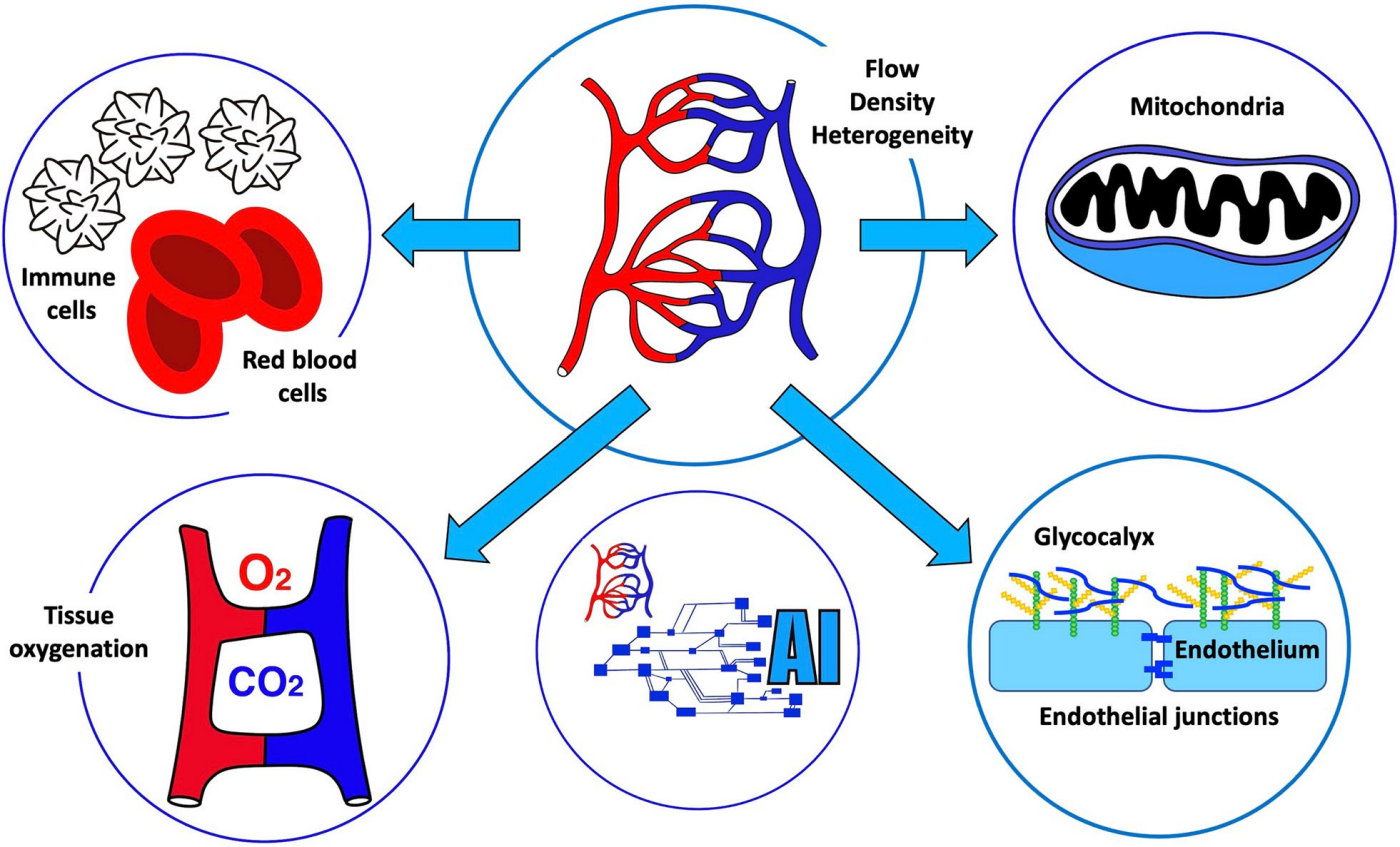
Integrative diagnostic platform: the future diagnostic platform will include hemodynamic components from macro to microcirculation, cellular and subcellular components and the immune function of cells. Artificial intelligence could assist in the development of algorithms and allow clinicians to make therapeutic decisions regarding the treatment of microcirculatory alterations

We can speculate on how the future diagnostic platform of the critically ill patient could be realized as technology develops, and more and more insight is gained into the pathogenesis and cellular origin of disease. Ultimately such a holistic diagnostic platform aimed at understanding the mechanism of disease and guiding therapy would have to encompass the total hierarchy of the cardiovascular system from the macrocirculation to the microcirculation including both cellular and subcellular components (Fig. 4). The various components of blood would also be integrated into this platform (Fig. 4). It is anticipated that HVM may include sensors and imaging modules, possibly even embedded into the tips of endoscopes or even digestible capsules, to observe the microcirculatory and cellular constituents in distant organs. The amount of information being generated continuously changing in time will be enormous. In this way, the system would create a virtual physiological model of the patient to allow for control of organ functions from the microcirculation down to the cells. Continuous surveillance of such a virtual patient would allow precise identification of (patho)physiological alterations in need of intervention. As technology progresses, future applications may include placing sensors and imaging modalities inside the patient for continuous monitoring of the variables known to control organ function, possibly in an automatically controlled loop manner. As advanced sensors and HVM imaging modalities develop and are placed in the patient, it may envision the creation of an ICU future with continuous in vivo monitoring [52].

Parallel to such hardware developments will be the development of an innovative mathematical resource that continuously develops physiological models of the virtual patient and has the ability to identify changes in the phenotype of organ and cellular systems. It is expected that AI will play a central role in the translation of the evaluation of the clinical condition of the virtual patient and to predict response to clinical interventions. This is achievable, for example, for the case of the microcirculation, by integrating AI methodologies with algorithmic analysis of microcirculatory images able to differentially diagnose specific alterations in the phenotype of the microcirculation known to be corrected by specific therapeutic interventions [53]. AI will allow to have a global vision of macrocirculatory and microcirculatory parameters and to better analyze their response to interventions. Ideally, AI may even suggest what might be the best intervention to implement taking into account the specificities of microcirculatory and microcirculatory alterations. AI could help us appreciate and test the coherence between macrocirculation and microcirculation. The insight provided into the functional state of the cardiovascular system using such a platform and aided by advanced machine learning algorithms and physiological models will provide targets for a more effective guidance of therapy of the critically ill patient.

## What are the future therapies targeting microcirculation in critical care?

The classical therapeutical interventions have variable effects. Fluids may improve microcirculation in the early stages of shock, but this improvement may not occur in the later stages [19]. The optimal amount of fluids administered remains difficult to determine, as an initial bolus may increase microvascular perfusion, whereas additional boluses may not affect microcirculation despite increased SV [20, 21]. On the other hand, excessive amounts of fluids and/or right ventricular dysfunction or high intrathoracic pressure are associated with venous stasis and glycocalyx damage, which further compromises microvascular perfusion [54]. The adequate amount of fluids required to resuscitate the microcirculation of a given patient is highly variable and remains difficult to determine.

Vasoactive agents also have variable effects on microcirculation, improving microcirculation in some patients but failing in others. It should always be kept in mind that the effect of vasopressors is dependent on blood volume, the functionality of vasopressor receptors and the intensity of microvascular alterations.

The development of new therapeutics is warranted to restore microcirculation when it is compromised.

Manipulation of nitric oxide (NO) pathways was one of the first routes explored given its crucial role in controlling microvascular perfusion [56]. In experimental septic shock, favorable results have been reported with the administration of l-arginine [63] and tetrahydrobiopterin (BH_4_) (cofactor of nitric oxide synthases) [57]. But studies that have tested the utility of direct or indirect nitric oxide augmentation in septic patients have not demonstrated improvement in sublingual microcirculation or organ dysfunction [59, 60].

Alternatively, manipulating the arachidonic pathway is an attractive future direction and trials evaluating the impact of vasodilatory prostaglandins are underway. Legrand et al. [51, 55] are currently conducting a multicenter, double-blind study, testing the impact of ilomedin (a prostacyclin analogue with vasodilatory and antithrombotic properties) on organ failure in septic shock patients with persistent microcirculation disorders (i.e., skin mottling or increased capillary refill time) despite hemodynamic optimization. This approach is extremely interesting, especially since in a recent multicenter, randomized clinical trial in COVID-19 adults with severe endotheliopathy, a 72 h infusion of prostacyclin (1 ng/kg/min) did not induce a statistically significant difference in the number of days of life without mechanical ventilation within 28 days; however, the point estimates favored the prostacyclin group in all analyses, including mortality and mean daily SOFA scores [56].

As during inflammatory and infectious states, cellular interactions within the microcirculation evolve toward a proadhesive and procoagulant phenotype, attempts to minimize cell aggregation should be tested. Multiple interventions were tested in experimental conditions, but few reached the clinical arena. Among these, ascorbate and several anticoagulants were particularly promising. Prior preclinical studies have repeatedly demonstrated that ascorbate improves microvascular perfusion and decrease white blood cells and platelets adhesion in experimental models of sepsis [57–59]. In septic patients, ascorbate also improved microvascular perfusion [60]. Due to the complex interaction between endothelial function, coagulation and inflammation, various anticoagulants have been tested. Activated protein C was the most promising agent, with clear demonstration of an improvement in microvascular perfusion both in experimental and clinical sepsis [61–63]. Other agents such as antithrombin or thrombomodulin also improved the microcirculation in experimental conditions [64, 65]. Interestingly, these papers showed not only an improvement in microvascular perfusion but also a reduction in aggregation and adhesion of white blood cells and platelets to the endothelium, a protection of the glycocalyx and a decrease in endothelial permeability (and thus vascular leakage), possibly through angiopoietin/TIE2 axis [66, 67]. However, The PROWESS-SHOCK study failed to confirm the benefit of activated protein C in sepsis [68], while the KyberSept study even showed harmful effects of antithrombin [69]. However, these molecules have not been administered with an individualized approach by limiting their use to patients with persistent microcirculatory alterations.

Because of its antioxidant properties, albumin is also an interesting therapeutic option to limit glycocalyx alterations and preserve endothelial function in intensive care patients [70]. However, the ALBIOS trial did not identify a significant benefit of albumin infusion in patients with sepsis [71]. However, a significant difference was observed in a post hoc analysis in patients with septic shock [71].

**Fig. 5:**
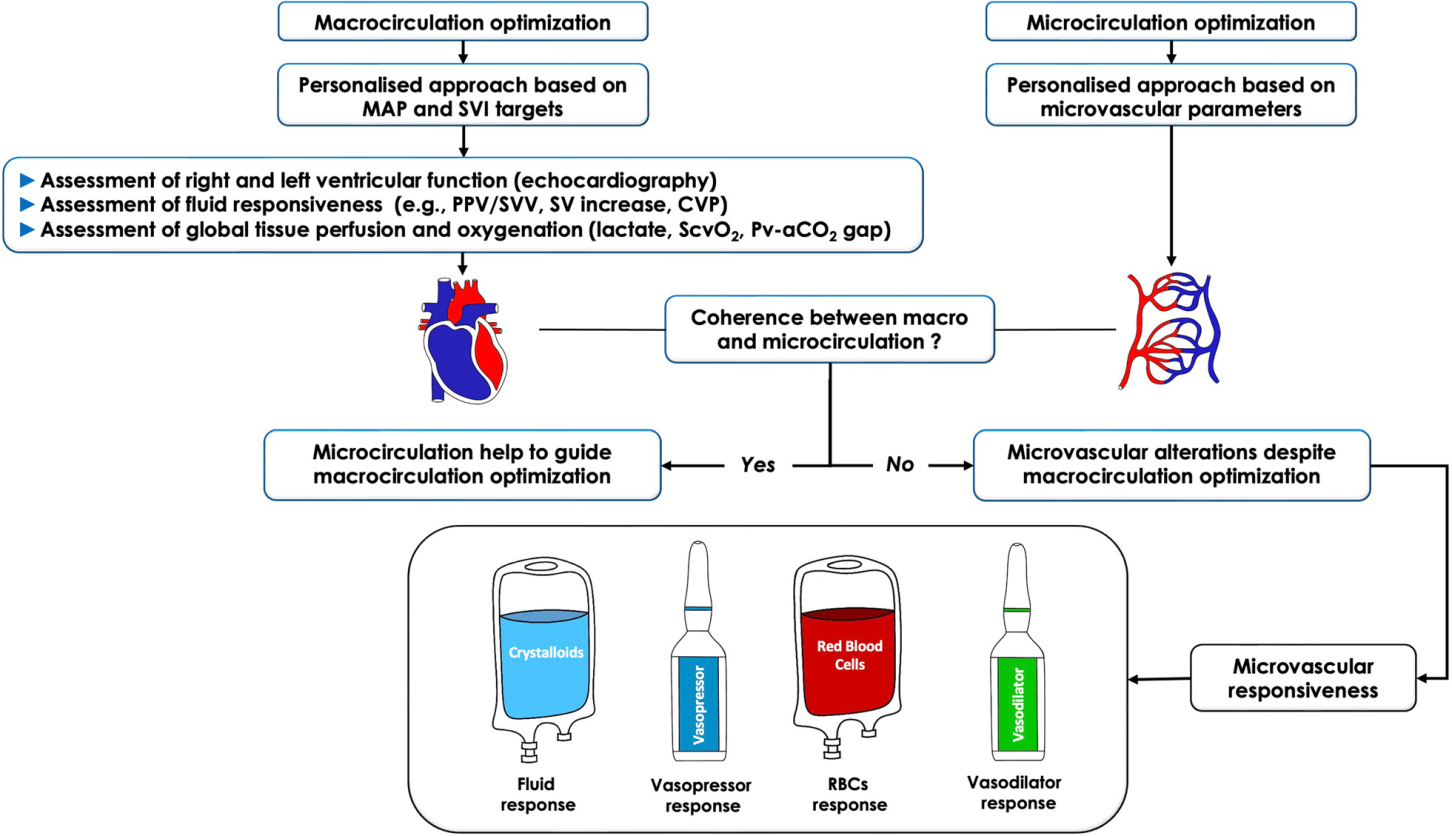
Proposal of an algorithm for the optimization of macro- and microcirculations. In the future, the hemodynamic optimization will have to individualize the microcirculation and the microcirculation. The concomitant evaluation of macro- and microhemodynamics will allow to test the coherence between macro- and microcirculation. In the absence of coherence between macro and microcirculation and in the face of persistent microvascular alterations despite macrovascular hemodynamic optimization, the microvascular response to the following therapeutic options can be tested taking into account the values of the microvascular parameters and the clinical context: 1 Performance of a fluid challenge. 2 Administration, or increase in doses, of vasopressors, or combination of another vasopressor (e.g., vasopressin if the primary vasopressor used is norepinephrine) (taking into account the individualized blood pressure level) if perfusion pressure is low. 3 Administration of packed red blood cells (especially if capillary density and/or microvascular hemoglobin is decreased). 4 In the future, strategies testing vasodilators or even antithrombotic molecules could be considered in case of persistence of microvascular alterations despite the previous therapeutic options and according to ongoing and future studies. PPV: Pulse pressure variation; SV: stroke volume; SVV: stroke volume variation; CVP: central venous pressure; ScvO_2_: central venous oxygen saturation; Pv-aCO_2_ gap: venous-to-arterial carbon dioxide difference. The order of interventions is indicative, and prioritization may vary according to patient. In each case, the effect of the interventions should be carefully checked

Future hemodynamic strategies in ICU patients should integrate macrocirculatory and microcirculatory optimization in an attempt to give clinicians the most complete picture of their patient's physiology and thus provide a clear path to treatment (Fig. 5). In the face of persistent microvascular alterations, clinicians should assess the microvascular response to a fluid challenge, and then in the absence of a response, test the administration and/or increase of vasopressor doses (with question about the optimal blood pressure level) (Fig. 5). The addition of packed RBC may be considered, especially in the face of decreased capillary density. In the future, the availability of capillary hemoglobin should make it possible to refine the administration of RBC. Finally, in the absence of response to previous therapeutic strategies, the administration of microvascular vasodilators may be considered in the future (Fig. 5). New algorithms should be tested in prospective randomized controlled trials on homogeneous populations of resuscitation patients at risk of microvascular alterations. Artificial intelligence could allow us to establish these algorithms and help the clinician to make therapeutic decisions regarding the treatment of microcirculatory alterations (Fig. 5).

## Conclusion

Hemodynamic management requires individualization of macrovascular and microvascular parameters. Clinicians are currently blind to what is happening in the microcirculation of organs, which prevents them from individualizing resuscitation by targeting the microcirculation. Limiting hemodynamic resuscitation to an optimization of the systemic hemodynamics without knowledge of the microcirculation exposes to persisting alterations in tissue perfusion or excessive therapeutic interventions. The challenge for the future is to have noninvasive, easy-to-use equipment that allows for reliable assessment and immediate quantitative analysis of the microcirculation at the patient's bedside. The use of automatic analysis and the future possibility of introducing artificial intelligence into the analysis software (e.g., in HVM-integrated software) could make it possible to eliminate observer bias and provide orientation of therapeutic options coupled with an analysis of the microvascular responses to the applied interventions. In addition, to gain caregiver confidence and support for the need to monitor the microcirculation, it is necessary to demonstrate that incorporating microcirculation analysis into the reasoning guiding hemodynamic resuscitation prevents organ dysfunction and improves the outcome of resuscitation patients.

## Data Availability

Not applicable.
